# Colistin-Resistant *mcr-1*–Positive Pathogenic *Escherichia coli* in Swine, Japan, 2007−2014

**DOI:** 10.3201/eid2207.160234

**Published:** 2016-07

**Authors:** Masahiro Kusumoto, Yoshitoshi Ogura, Yasuhiro Gotoh, Taketoshi Iwata, Tetsuya Hayashi, Masato Akiba

**Affiliations:** National Institute of Animal Health, National Agriculture and Food Research Organization, Ibaraki, Japan (M. Kusumoto, T. Iwata, M. Akiba);; Kyushu University, Fukuoka, Japan (Y. Ogura, T. Hayashi);; University of Miyazaki, Miyazaki, Japan (Y. Gotoh); Osaka Prefecture University, Osaka, Japan (M. Akiba)

**Keywords:** colistin resistance, mcr-1, pathogenic Escherichia coli, swine, Japan, bacteria, antimicrobial resistance

**To the Editor:** Colistin is an old-generation antimicrobial agent; however, because it is one of the few agents that remain effective against multidrug-resistant gram-negative bacteria (e.g., carbapenem-resistant *Pseudomonas aeruginosa* and *Enterobacteriaceae*), its clinical usefulness is being increasingly recognized (*1*). Previous reports have described the mechanisms of colistin resistance (*2*) as being chromosomally mediated and not associated with horizontal gene transfer. However, from 2011 through 2014, a plasmid-encoded colistin-resistance gene, *mcr-1*, was identified in colistin-resistant *Escherichia coli* isolated in China, particularly from animals. Specifically, *mcr-1*–positive isolates were found in 21% of healthy swine at slaughter, 15% of marketed pork and chicken meat, and 1% of hospitalized human patients (*3*). A study of *E. coli* isolated from healthy cattle, swine, and chickens in Japan during 2000–2014 found only 2 (0.02%) of 9,308 isolates positive for *mcr-1* (*4*). We report the rates at which *mcr-1* was detected in our stored collection of *E. coli* isolates from diseased swine (swine with diarrhea or edema disease), hereafter referred to as swine-pathogenic *E. coli*.

We recently analyzed swine-pathogenic *E. coli* strains isolated from diseased swine throughout Japan during 1991–2014 (*5*). We analyzed all swine disease-associated *E. coli* strains isolated from the 23 Livestock Hygiene Service Centers in Japan (including prefectures that covered 75% of total swine production in Japan in 2014) and sent to the National Institute of Animal Health for diagnostic purposes during 1991–2014. Among the 967 strains examined, 684 (71%) belonged to *E. coli* serogroup O139, O149, O116, or OSB9. 

In the study reported here, we investigated these 684 strains for susceptibility to colistin and for *mcr-1* carriage. The strains from the 4 predominant serogroups (Technical Appendix Table) can be considered representative of swine-pathogenic *E. coli* strains isolated from farm animals, but not food products, in Japan. MICs were determined by using the agar dilution method according to the recommendations of the Clinical and Laboratory Standards Institute (*6*). The presence of *mcr-1* was detected by PCR (*3*).

Among the 684 strains examined, colistin MICs exhibited a bimodal distribution of 0.25–128 μg/mL and peaked at 0.5 and 16 μg/mL (Technical Appendix Figure). According to the European Committee on Antimicrobial Susceptibility Testing criterion (*7*), in which isolates with an MIC of >4 μg/mL are considered colistin resistant, 309 (45%) of the 684 strains were classified as colistin resistant. The gene *mcr-1* was detected in 90 (13%) strains, and the MICs for these *mcr-1*–positive strains ranged from 8 to 128 μg/mL (Technical Appendix Figure). Among the 309 colistin-resistant strains, *mcr-1*–positive and *mcr-1*–negative isolates had the same 50% and 90% MICs, 16 and 32 μg/mL, respectively. These results indicate that a high proportion of swine-pathogenic *E. coli* in Japan are resistant to colistin, that *mcr-1* has already been widely disseminated among these strains, and that the level of colistin resistance mediated by *mcr-1* is similar to that mediated by *mcr-1*–independent mechanisms.

In 2004, colistin-resistant *E. coli* already represented 77% of the isolates, and the positivity rates varied from year to year (26%–82%) (Figure). First detection of *mcr-1*–positive strains was in 2007, and the proportion of *mcr-1* positivity has risen, especially since 2009 (Figure). During 2013–2014, approximately half of the strains isolated were *mcr-1* positive (Figure), and most colistin-resistant strains isolated during these 2 years carried *mcr-1* (85% and 62% in 2013 and 2014, respectively). Of note, the rates of *mcr-1*–positive strains among the 4 serogroups isolated from 2010 through 2014 did not differ significantly (χ^2^ test): 22 (20%) of 110 in O139, 38 (38%) of 100 in O149, 19 (26%) of 73 in O116, and 6 (32%) of 19 in OSB9. This finding suggests that the sharp rise in the proportion of *mcr-1*–positive strains has been driven by plasmid-mediated horizontal gene transfer, not by the expansion of a specific clone. 

**Figure:**
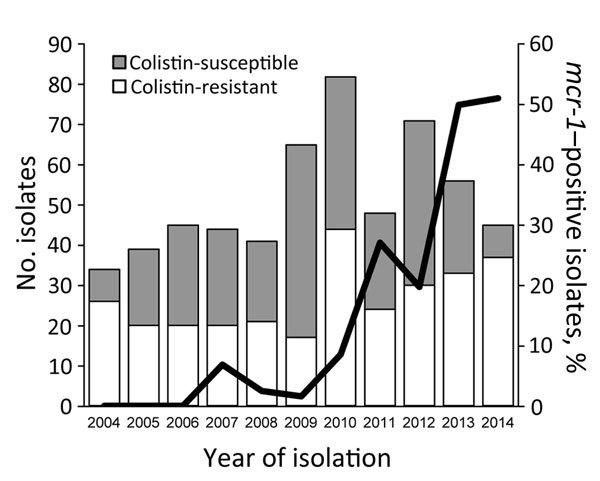
Changes in the numbers of colistin-susceptible and colistin-resistant *Escherichia coli* isolated from swine with diarrhea or edema disease, Japan, 2004–2014. The line shows the changes in proportion of *mcr-1*–positive isolates among the total isolates for each year.

In Japan, rates of isolation of colistin-resistant and *mcr-1*–positive *E. coli* strains from healthy animals are low, 1.00% and 0.02% of 9,308 strains examined, respectively (*4*). These low rates may be the result of the prudent use of colistin in Japan. During 2000–2007 in Japan, colistin use in swine did not increase significantly (*8*). However, our data show that *mcr-1* has recently been disseminated among swine-pathogenic *E. coli* in Japan, which might be associated with the use of colistin to treat disease in swine. Although *mcr-1*–positive bacteria have not yet been isolated from humans in Japan (*4*), the sharp increase in swine-pathogenic *E. coli* in animal strains implies a risk for transmission of *mcr-1* from these strains to human-pathogenic bacteria, a serious concern for human medicine. More active surveillance of *mcr-1*–positive colistin-resistant bacteria in human and animal environments is needed.

Technical Appendix Information about isolates used in study of *mcr-1*–positive colistin-resistant pathogenic *Escherichia coli* in swine, Japan, 2007–2014, and MICs of colistin for *E. coli* isolated from diseased swine in Japan 1991–2014.
