# Supplementary material for: Colistin-Resistant mcr-1–Positive Pathogenic Escherichia coli in Swine, Japan, 2007−2014
Source: Emerg Infect Dis. 2016 Jul;22(7):1315–7. doi: 10.3201/eid2207.160234 (PMC4918142; doi:10.3201/eid2207.160234)
Supplement: Technical Appendix — Information about isolates used in study of mcr-1–positive colistin-resistant pathogenic Escherichia coli in swine, Japan, 2007–2014, and MICs of colistin for E. coli isolated from diseased swine in Japan 1991–2014. [file 16-0234-Techapp-s1.pdf]

Article DOI: http://dx.doi.org/10.3201/eid2207.160234

Colistin-Resistant *mcr-1*–Positive Pathogenic *Escherichia coli* in Swine, Japan, 2007–2014

Technical Appendix

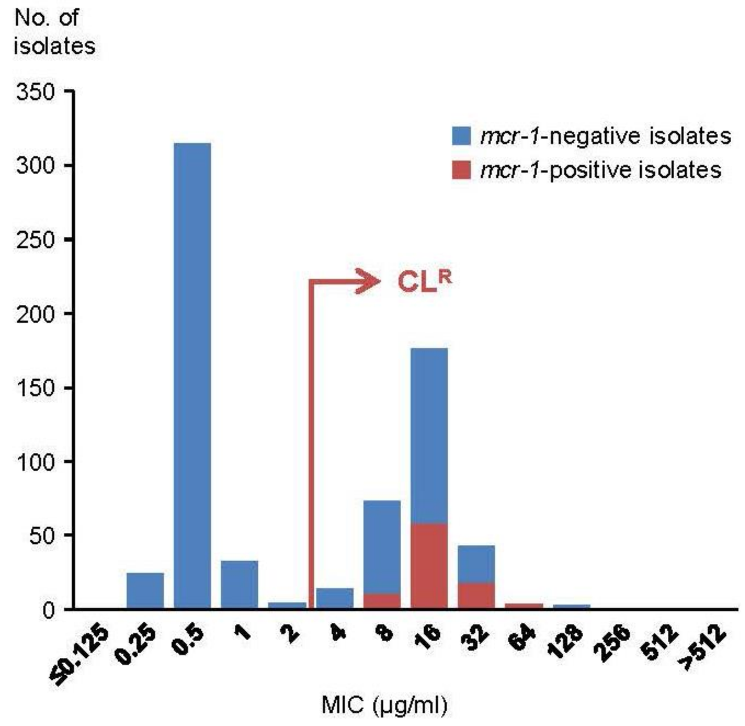

Technical Appendix Figure. Distribution of the MICs of colistin for the 684 *E. coli* isolates from diseased swine in Japan between 1991 and 2014. Because the breakpoint MIC of colistin for Enterobacteriaceae has not yet been published by the CLSI (*1*), isolates with an MIC of ≥4 µg/ml were considered colistin resistant according to the EUCAST (*2*) criteria, as indicated by the red arrow. The numbers of isolates presenting each MIC are indicated with bars; *mcr-1*-negative and *mcr-1*-positive isolates are shown in blue and red, respectively. There were three and two isolates with MICs of 64 and 128 µg/ml, respectively. The MICs of colistin for the *E. coli* ATCC 25922 and *Pseudomonas aeruginosa* ATCC 27853 reference strains (0.5 and 1 µg/mL, respectively) were within the quality control ranges established by CLSI (0.25−2 and 0.5−4 µg/mL, respectively) (*1*).

References

1. Clinical and Laboratory Standards Institute. Performance standards for antimicrobial susceptibility testing; 24th informational supplement. Document M100–S24: Wayne (PA): The Institute; 2014.

2. European Committee on Antimicrobial Susceptibility Testing. Breakpoint tables for interpretation of MICs and zone diameters. Version 60 [cited 2016 Feb 6]. http://www.eucast.org/clinical_breakpoints/

Technical Appendix Table. Information of *E. coli* isolates used in study of *mcr-1*-Positive Colistin-Resistant Pathogenic *Escherichia coli* in Swine, Japan, 2007–2014

Strain name	*mcr-1* carriage*	Susceptibility to colistin		O serogroup‡	Characteristic features of isolates		
		MIC (g/mL)	Resistance†		Year	Prefecture	Reported case
E1125	+	128	R	O149	2013	T	Diarrhea
E1410	−	128	R	O116	2011	D	Diarrhea
E0610	+	64	R	O116	2012	L	Diarrhea
E0611	+	64	R	O116	2012	L	Diarrhea
E1046	+	64	R	O149	2012	H	Diarrhea
E0107	+	32	R	O116	2008	D	Diarrhea
E0658	+	32	R	O139	2010	G	Edema disease
E0912	+	32	R	O139	2013	B	Edema disease
E0924	+	32	R	O149	2013	B	Diarrhea
E0961	+	32	R	O139	2013	P	Edema disease
E1037	+	32	R	O116	2011	H	Diarrhea
E1043	+	32	R	O149	2011	H	Diarrhea
E1065	+	32	R	O149	2013	H	Diarrhea
E1128	+	32	R	O116	2013	T	Diarrhea
E1129	+	32	R	O116	2013	T	Diarrhea
E1131	+	32	R	O116	2014	T	Diarrhea
E1264	+	32	R	O149	2014	I	Diarrhea
E1266	+	32	R	O149	2014	E	Diarrhea
E1267	+	32	R	O149	2014	E	Diarrhea
E1268	+	32	R	O149	2014	E	Diarrhea
E1314	+	32	R	O149	2014	I	Diarrhea
E1401	+	32	R	O116	2011	D	Diarrhea
E1514	+	32	R	O139	2013	P	Edema disease
E0057	−	32	R	O139	2010	M	Diarrhea
E0254	−	32	R	O139	2007	S	Edema disease
E0383	−	32	R	O116	2005	F	Diarrhea
E0389	−	32	R	O116	2006	F	Diarrhea
E0392	−	32	R	O116	2008	F	Diarrhea
E0393	−	32	R	O116	2008	F	Diarrhea
E0400	−	32	R	O116	2009	F	Diarrhea
E0517	−	32	R	O139	2005	C	Edema disease
E0546	−	32	R	O139	2000	F	Edema disease
E0881	−	32	R	O139	2004	B	Edema disease
E0882	−	32	R	O139	2004	B	Edema disease
E0883	−	32	R	O139	2004	B	Edema disease
E0884	−	32	R	O139	2004	B	Edema disease
E1057	−	32	R	O139	2012	H	Edema disease
E1213	−	32	R	OSB9	2005	I	Diarrhea
E1214	−	32	R	OSB9	2006	I	Diarrhea
E1271	−	32	R	O116	2014	E	Diarrhea
E1272	−	32	R	O116	2014	E	Diarrhea
E1274	−	32	R	O116	2014	E	Diarrhea
E1275	−	32	R	O116	2014	E	Diarrhea
E1277	−	32	R	O116	2014	E	Diarrhea
E1278	−	32	R	O116	2014	E	Diarrhea
E1280	−	32	R	O116	2014	E	Diarrhea
E1282	−	32	R	O116	2014	E	Diarrhea
E0103	+	16	R	O116	2007	D	Diarrhea
E0105	+	16	R	O116	2007	D	Diarrhea
E0403	+	16	R	O116	2010	F	Diarrhea
E0404	+	16	R	O116	2011	F	Diarrhea

Strain name	*mcr-1* carriage*	Susceptibility to colistin		O serogroup‡	Characteristic features of isolates		
		MIC (g/mL)	Resistance†		Year	Prefecture	Reported case
E0569	+	16	R	O149	2012	F	Diarrhea
E0662	+	16	R	O149	2011	G	Diarrhea
E0663	+	16	R	O149	2012	G	Diarrhea
E0664	+	16	R	O149	2012	G	Diarrhea
E0669	+	16	R	O149	2012	G	Diarrhea
E0685	+	16	R	O139	2010	G	Edema disease
E0688	+	16	R	O139	2010	G	Edema disease
E0692	+	16	R	O149	2011	G	Diarrhea
E0693	+	16	R	O149	2011	G	Diarrhea
E0694	+	16	R	O149	2011	G	Diarrhea
E0695	+	16	R	O149	2011	G	Diarrhea
E0696	+	16	R	O149	2012	G	Diarrhea
E0697	+	16	R	O149	2012	G	Diarrhea
E0714	+	16	R	O139	2013	E	Edema disease
E0715	+	16	R	O139	2013	E	Edema disease
E0716	+	16	R	O139	2013	E	Edema disease
E0717	+	16	R	O139	2013	E	Edema disease
E0908	+	16	R	O139	2013	B	Edema disease
E0909	+	16	R	O139	2013	B	Edema disease
E0910	+	16	R	O139	2013	B	Edema disease
E0911	+	16	R	O139	2013	B	Edema disease
E0938	+	16	R	O116	2013	B	Diarrhea
E0962	+	16	R	O139	2013	P	Edema disease
E1036	+	16	R	O149	2011	H	Diarrhea
E1052	+	16	R	O116	2012	H	Diarrhea
E1053	+	16	R	O116	2012	H	Diarrhea
E1062	+	16	R	O149	2013	H	Diarrhea
E1070	+	16	R	O149	2013	H	Diarrhea
E1104	+	16	R	O139	2010	J	Edema disease
E1122	+	16	R	O149	2013	T	Diarrhea
E1145	+	16	R	O139	2011	W	Edema disease
E1190	+	16	R	O139	2013	A	Edema disease
E1191	+	16	R	O139	2013	A	Edema disease
E1221	+	16	R	OSB9	2010	I	Diarrhea
E1235	+	16	R	O139	2013	I	Diarrhea
E1265	+	16	R	O149	2014	I	Diarrhea
E1310	+	16	R	O149	2014	I	Diarrhea
E1337	+	16	R	O116	2014	N	Diarrhea
E1346	+	16	R	OSB9	2014	J	Edema disease
E1348	+	16	R	OSB9	2014	J	Edema disease
E1349	+	16	R	OSB9	2014	J	Edema disease
E1350	+	16	R	OSB9	2014	J	Edema disease
E1409	+	16	R	O116	2011	D	Diarrhea
E1422	+	16	R	O116	2012	D	Diarrhea
E1516	+	16	R	O149	2014	U	Diarrhea
E1517	+	16	R	O149	2014	U	Diarrhea
E1518	+	16	R	O149	2014	U	Diarrhea
E1519	+	16	R	O149	2014	U	Diarrhea
E1520	+	16	R	O149	2014	U	Diarrhea
E1521	+	16	R	O149	2014	U	Diarrhea
E1522	+	16	R	O149	2014	U	Diarrhea
E1523	+	16	R	O149	2014	U	Diarrhea
E1524	+	16	R	O149	2014	U	Diarrhea
E1525	+	16	R	O149	2014	U	Diarrhea
E0002	−	16	R	O139	2007	I	Edema disease
E0003	−	16	R	O139	2007	I	Edema disease
E0005	−	16	R	O139	2008	I	Edema disease
E0006	−	16	R	O139	2009	I	Edema disease
E0007	−	16	R	O139	2009	I	Edema disease
E0043	−	16	R	O139	2010	M	Diarrhea
E0044	−	16	R	O139	2010	M	Diarrhea
E0045	−	16	R	O139	2010	M	Diarrhea
E0046	−	16	R	O139	2010	M	Diarrhea
E0047	−	16	R	O139	2010	M	Diarrhea
E0048	−	16	R	O139	2010	M	Diarrhea
E0049	−	16	R	O139	2010	M	Diarrhea
E0050	−	16	R	O139	2010	M	Diarrhea
E0051	−	16	R	O139	2010	M	Diarrhea

Strain name	*mcr-1* carriage*	Susceptibility to colistin MIC (g/mL)	Resistance†	O serogroup‡	Characteristic features of isolates Year	Prefecture	Reported case
E0052	−	16	R	O139	2010	M	Diarrhea
E0053	−	16	R	O139	2010	M	Diarrhea
E0054	−	16	R	O139	2010	M	Diarrhea
E0055	−	16	R	O139	2010	M	Diarrhea
E0056	−	16	R	O139	2010	M	Diarrhea
E0058	−	16	R	O139	2010	M	Diarrhea
E0059	−	16	R	O139	2010	M	Diarrhea
E0124	−	16	R	O139	2009	D	Diarrhea
E0217	−	16	R	O139	2010	L	Diarrhea
E0218	−	16	R	O139	2010	L	Diarrhea
E0219	−	16	R	O139	2010	L	Diarrhea
E0220	−	16	R	O139	2010	L	Diarrhea
E0234	−	16	R	O139	1991	S	Edema disease
E0237	−	16	R	O139	1992	S	Edema disease
E0250	−	16	R	O139	2004	S	Edema disease
E0251	−	16	R	O139	2005	S	Edema disease
E0252	−	16	R	O139	2005	S	Edema disease
E0259	−	16	R	O139	2005	S	Edema disease
E0260	−	16	R	O139	2005	S	Edema disease
E0408	−	16	R	O139	2004	F	Diarrhea
E0414	−	16	R	O139	2007	F	Diarrhea
E0449	−	16	R	O139	2005	C	Edema disease
E0459	−	16	R	OSB9	2006	C	Diarrhea
E0460	−	16	R	OSB9	2006	C	Diarrhea
E0461	−	16	R	OSB9	2006	C	Diarrhea
E0540	−	16	R	O139	1996	F	Edema disease
E0543	−	16	R	O139	1998	F	Edema disease
E0550	−	16	R	O139	2006	F	Edema disease
E0587	−	16	R	O139	2012	L	Edema disease
E0588	−	16	R	O139	2012	L	Edema disease
E0633	−	16	R	O116	2007	G	Diarrhea
E0635	−	16	R	O116	2007	G	Diarrhea
E0636	−	16	R	O116	2007	G	Diarrhea
E0637	−	16	R	O116	2007	G	Diarrhea
E0638	−	16	R	O116	2007	G	Diarrhea
E0643	−	16	R	O116	2007	G	Diarrhea
E0673	−	16	R	O139	2005	R	Edema disease
E0677	−	16	R	O116	2007	G	Diarrhea
E0686	−	16	R	O139	2010	G	Edema disease
E0687	−	16	R	O139	2010	G	Edema disease
E0866	−	16	R	O139	1995	B	Edema disease
E0867	−	16	R	O139	1995	B	Edema disease
E0871	−	16	R	O139	2002	B	Edema disease
E0872	−	16	R	O139	2002	B	Edema disease
E0874	−	16	R	O139	2004	B	Edema disease
E0875	−	16	R	O139	2004	B	Edema disease
E0876	−	16	R	O139	2004	B	Edema disease
E0877	−	16	R	O139	2004	B	Edema disease
E0878	−	16	R	O139	2004	B	Edema disease
E0879	−	16	R	O139	2004	B	Edema disease
E0880	−	16	R	O139	2004	B	Edema disease
E0888	−	16	R	O139	2005	B	Edema disease
E0889	−	16	R	O139	2005	B	Edema disease
E0890	−	16	R	O139	2006	B	Edema disease
E0891	−	16	R	O139	2006	B	Edema disease
E0892	−	16	R	O139	2006	B	Edema disease
E0893	−	16	R	O139	2008	B	Edema disease
E0894	−	16	R	O139	2008	B	Edema disease
E0898	−	16	R	O139	2009	B	Diarrhea
E0901	−	16	R	O139	2011	B	Edema disease
E0902	−	16	R	O139	2011	B	Edema disease
E0903	−	16	R	O139	2011	B	Edema disease
E0906	−	16	R	O139	2013	B	Edema disease
E0907	−	16	R	O139	2013	B	Edema disease
E0920	−	16	R	O149	2004	B	Diarrhea
E0921	−	16	R	O149	2004	B	Diarrhea
E0922	−	16	R	O149	2004	B	Diarrhea
E0923	−	16	R	O149	2004	B	Diarrhea

Strain name	*mcr-1* carriage*	Susceptibility to colistin MIC (g/mL)	Resistance†	O serogroup‡	Characteristic features of isolates Year	Prefecture	Reported case
E0975	−	16	R	OSB9	2005	H	Diarrhea
E0987	−	16	R	O139	2006	H	Edema disease
E1055	−	16	R	O149	2012	H	Diarrhea
E1066	−	16	R	O149	2013	H	Diarrhea
E1091	−	16	R	O139	2006	J	Edema disease
E1093	−	16	R	O139	2006	J	Edema disease
E1095	−	16	R	O139	2007	J	Edema disease
E1099	−	16	R	O139	2008	J	Edema disease
E1101	−	16	R	O139	2009	J	Edema disease
E1102	−	16	R	O139	2009	J	Edema disease
E1103	−	16	R	O139	2009	J	Edema disease
E1109	−	16	R	O139	2014	J	Edema disease
E1110	−	16	R	O139	2014	J	Edema disease
E1114	−	16	R	OSB9	2010	T	Diarrhea
E1187	−	16	R	O139	2012	A	Edema disease
E1188	−	16	R	O139	2012	A	Edema disease
E1206	−	16	R	OSB9	2003	I	Diarrhea
E1210	−	16	R	OSB9	2004	I	Diarrhea
E1211	−	16	R	OSB9	2004	I	Diarrhea
E1212	−	16	R	OSB9	2004	I	Diarrhea
E1220	−	16	R	O139	2009	I	Edema disease
E1222	−	16	R	OSB9	2011	I	Diarrhea
E1225	−	16	R	O139	2012	I	Edema disease
E1241	−	16	R	O139	2003	I	Edema disease
E1253	−	16	R	O139	1998	I	Edema disease
E1257	−	16	R	O139	1998	I	Edema disease
E1258	−	16	R	O139	2004	I	Edema disease
E1259	−	16	R	O139	2004	I	Edema disease
E1269	−	16	R	O139	2013	E	Edema disease
E1270	−	16	R	O139	2013	E	Edema disease
E1273	−	16	R	O116	2014	E	Diarrhea
E1276	−	16	R	O116	2014	E	Diarrhea
E1279	−	16	R	O116	2014	E	Diarrhea
E1281	−	16	R	O116	2014	E	Diarrhea
E1385	−	16	R	O116	2010	D	Diarrhea
E1388	−	16	R	O116	2010	D	Diarrhea
E0951	+	8	R	O139	2012	V	Edema disease
E0993	+	8	R	O149	2007	H	Diarrhea
E1012	+	8	R	OSB9	2009	H	Diarrhea
E1059	+	8	R	OSB9	2012	H	Diarrhea
E1127	+	8	R	O149	2013	T	Diarrhea
E1143	+	8	R	O116	2011	W	Diarrhea
E1232	+	8	R	O116	2013	I	Diarrhea
E1233	+	8	R	O116	2013	I	Diarrhea
E1391	+	8	R	O139	2010	D	Edema disease
E1443	+	8	R	O116	2013	D	Diarrhea
E0004	−	8	R	O139	2009	I	Edema disease
E0008	−	8	R	O139	2009	I	Edema disease
E0095	−	8	R	O139	2006	D	Edema disease
E0099	−	8	R	O116	2007	D	Diarrhea
E0391	−	8	R	O116	2008	F	Diarrhea
E0398	−	8	R	O116	2009	F	Diarrhea
E0462	−	8	R	OSB9	2006	C	Diarrhea
E0464	−	8	R	OSB9	2007	C	Diarrhea
E0478	−	8	R	O149	2008	C	Diarrhea
E0479	−	8	R	O149	2008	C	Diarrhea
E0507	−	8	R	O149	2010	C	Diarrhea
E0508	−	8	R	O149	2010	C	Diarrhea
E0509	−	8	R	O149	2010	C	Diarrhea
E0510	−	8	R	O149	2010	C	Diarrhea
E0511	−	8	R	O149	2010	C	Diarrhea
E0512	−	8	R	O149	2010	C	Diarrhea
E0513	−	8	R	O149	2010	C	Diarrhea
E0535	−	8	R	O139	2006	O	Edema disease
E0548	−	8	R	O139	2005	F	Edema disease
E0565	−	8	R	O149	2005	F	Diarrhea
E0634	−	8	R	O116	2007	G	Diarrhea
E0639	−	8	R	O116	2007	G	Diarrhea

	mcr-1	Susceptibility to colistin			Characteristic features of isolates		
Strain name	carriage*	MIC (g/mL)	Resistance†	O serogroup‡	Year	Prefecture	Reported case
E0640	−	8	R	O116	2007	G	Diarrhea
E0675	−	8	R	O139	2005	R	Edema disease
E0678	−	8	R	O116	2008	G	Diarrhea
E0733	−	8	R	O116	2006	E	Diarrhea
E0742	−	8	R	O116	2006	E	Diarrhea
E0743	−	8	R	O116	2006	E	Diarrhea
E0744	−	8	R	O116	2006	E	Diarrhea
E0873	−	8	R	O139	2004	B	Edema disease
E0885	−	8	R	O139	2005	B	Edema disease
E0886	−	8	R	O139	2005	B	Edema disease
E0887	−	8	R	O139	2005	B	Edema disease
E0895	−	8	R	O139	2008	B	Edema disease
E0896	−	8	R	O139	2008	B	Edema disease
E0897	−	8	R	O139	2008	B	Edema disease
E0940	−	8	R	O139	2008	V	Edema disease
E0959	−	8	R	O139	2012	P	Edema disease
E0960	−	8	R	O139	2012	P	Edema disease
E1019	−	8	R	O149	2010	H	Diarrhea
E1040	−	8	R	O149	2011	H	Diarrhea
E1041	−	8	R	O149	2011	H	Diarrhea
E1097	−	8	R	O139	2008	J	Edema disease
E1100	−	8	R	O139	2009	J	Edema disease
E1106	−	8	R	O139	2010	J	Edema disease
E1107	−	8	R	O139	2010	J	Edema disease
E1108	−	8	R	O139	2011	J	Edema disease
E1113	−	8	R	O139	2010	T	Edema disease
E1115	−	8	R	O139	2011	T	Edema disease
E1116	−	8	R	O149	2011	T	Diarrhea
E1180	−	8	R	O139	2012	A	Edema disease
E1183	−	8	R	O139	2012	A	Edema disease
E1184	−	8	R	O139	2012	A	Edema disease
E1185	−	8	R	O139	2012	A	Edema disease
E1186	−	8	R	O139	2012	A	Edema disease
E1209	−	8	R	OSB9	2004	I	Diarrhea
E1215	−	8	R	OSB9	2006	I	Diarrhea
E1243	−	8	R	O139	2003	I	Edema disease
E1245	−	8	R	O139	2003	I	Edema disease
E1252	−	8	R	O149	1997	I	Diarrhea
E1254	−	8	R	O149	1997	I	Diarrhea
E1260	−	8	R	O149	2004	I	Diarrhea
E1303	−	8	R	O139	2012	I	Others/unknown
E0117	−	4	R	O116	2009	D	Diarrhea
E0549	−	4	R	O139	2005	F	Edema disease
E0648	−	4	R	O116	2008	G	Diarrhea
E0674	−	4	R	O139	2005	R	Edema disease
E0679	−	4	R	O116	2008	G	Diarrhea
E0680	−	4	R	O116	2008	G	Diarrhea
E0681	−	4	R	O116	2008	G	Diarrhea
E0682	−	4	R	O116	2008	G	Diarrhea
E0737	−	4	R	O149	2004	E	Diarrhea
E1035	−	4	R	O149	2011	H	Diarrhea
E1056	−	4	R	O149	2012	H	Diarrhea
E1262	−	4	R	O149	2009	I	Diarrhea
E1296	−	4	R	O149	2009	I	Diarrhea
E0131	−	2	S	O139	2010	D	Diarrhea
E0672	−	2	S	O139	2004	R	Edema disease
E0969	−	2	S	O149	2003	H	Diarrhea
E0970	−	2	S	O149	2003	H	Diarrhea
E0022	−	1	S	O139	2009	A	Edema disease
E0125	−	1	S	O116	2009	D	Diarrhea
E0226	−	1	S	O149	2005	S	Diarrhea
E0233	−	1	S	O149	2007	S	Diarrhea
E0387	−	1	S	O116	2006	F	Diarrhea
E0409	−	1	S	O139	2004	F	Diarrhea
E0465	−	1	S	OSB9	2007	C	Diarrhea
E0467	−	1	S	OSB9	2007	C	Diarrhea
E0468	−	1	S	OSB9	2007	C	Diarrhea
E0469	−	1	S	OSB9	2007	C	Diarrhea

Strain name	*mcr-1* carriage*	Susceptibility to colistin		O serogroup‡	Characteristic features of isolates		
		MIC (g/mL)	Resistance†		Year	Prefecture	Reported case
E0470	−	1	S	OSB9	2007	C	Diarrhea
E0471	−	1	S	OSB9	2007	C	Diarrhea
E0472	−	1	S	OSB9	2007	C	Diarrhea
E0475	−	1	S	OSB9	2008	C	Diarrhea
E0477	−	1	S	O149	2008	C	Diarrhea
E0481	−	1	S	O139	2009	C	Edema disease
E0490	−	1	S	O149	2009	C	Diarrhea
E0498	−	1	S	OSB9	2010	C	Diarrhea
E0499	−	1	S	OSB9	2010	C	Diarrhea
E0502	−	1	S	O139	2008	C	Edema disease
E0505	−	1	S	OSB9	2010	C	Diarrhea
E0506	−	1	S	OSB9	2010	C	Diarrhea
E0514	−	1	S	OSB9	2010	C	Diarrhea
E0520	−	1	S	OSB9	2007	C	Diarrhea
E1061	−	1	S	O149	2013	H	Diarrhea
E1223	−	1	S	O116	2011	I	Diarrhea
E1237	−	1	S	O149	2014	I	Diarrhea
E1389	−	1	S	O116	2010	D	Diarrhea
E1395	−	1	S	O116	2011	D	Diarrhea
E1402	−	1	S	O116	2011	D	Diarrhea
E1415	−	1	S	O149	2012	D	Diarrhea
E1454	−	1	S	O116	2014	D	Diarrhea
E0001	−	0.5	S	O139	2007	I	Edema disease
E0009	−	0.5	S	O139	2009	A	Edema disease
E0010	−	0.5	S	O139	2009	A	Edema disease
E0011	−	0.5	S	O139	2009	A	Edema disease
E0017	−	0.5	S	O139	2009	A	Edema disease
E0018	−	0.5	S	O139	2009	A	Edema disease
E0019	−	0.5	S	O139	2009	A	Edema disease
E0020	−	0.5	S	O139	2009	A	Edema disease
E0021	−	0.5	S	O139	2009	A	Edema disease
E0023	−	0.5	S	O139	2009	A	Edema disease
E0024	−	0.5	S	O139	2009	A	Edema disease
E0025	−	0.5	S	O139	2009	A	Edema disease
E0026	−	0.5	S	O139	2009	A	Edema disease
E0027	−	0.5	S	O139	2009	A	Edema disease
E0092	−	0.5	S	O149	2006	D	Diarrhea
E0098	−	0.5	S	O149	2007	D	Diarrhea
E0101	−	0.5	S	O116	2007	D	Diarrhea
E0102	−	0.5	S	O116	2007	D	Diarrhea
E0108	−	0.5	S	O116	2008	D	Diarrhea
E0110	−	0.5	S	O116	2008	D	Diarrhea
E0111	−	0.5	S	O139	2008	D	Edema disease
E0113	−	0.5	S	O116	2009	D	Diarrhea
E0115	−	0.5	S	O139	2009	D	Others/unknown
E0116	−	0.5	S	O116	2009	D	Diarrhea
E0121	−	0.5	S	O116	2009	D	Diarrhea
E0126	−	0.5	S	O149	2009	D	Others/unknown
E0127	−	0.5	S	O116	2010	D	Diarrhea
E0128	−	0.5	S	O139	2010	D	Edema disease
E0129	−	0.5	S	O149	2010	D	Others/unknown
E0132	−	0.5	S	O116	2010	D	Diarrhea
E0221	−	0.5	S	O149	1993	S	Diarrhea
E0222	−	0.5	S	O149	1993	S	Diarrhea
E0223	−	0.5	S	O149	1994	S	Diarrhea
E0224	−	0.5	S	O149	1994	S	Diarrhea
E0225	−	0.5	S	O149	1995	S	Diarrhea
E0227	−	0.5	S	O149	2004	S	Diarrhea
E0228	−	0.5	S	O149	2004	S	Diarrhea
E0229	−	0.5	S	O149	2005	S	Diarrhea
E0230	−	0.5	S	O149	2005	S	Diarrhea
E0231	−	0.5	S	O149	2006	S	Diarrhea
E0232	−	0.5	S	O149	2006	S	Diarrhea
E0238	−	0.5	S	O139	1993	S	Diarrhea
E0241	−	0.5	S	O139	1995	S	Edema disease
E0243	−	0.5	S	O139	1997	S	Diarrhea
E0258	−	0.5	S	O139	2005	S	Edema disease
E0348	−	0.5	S	O149	1993	K	Diarrhea

Strain name	*mcr-1* carriage*	Susceptibility to colistin		O serogroup‡	Characteristic features of isolates		
		MIC (g/mL)	Resistance†		Year	Prefecture	Reported case
E0349	−	0.5	S	O149	1993	K	Diarrhea
E0350	−	0.5	S	O149	1994	K	Diarrhea
E0351	−	0.5	S	O149	1994	K	Diarrhea
E0352	−	0.5	S	O149	1994	K	Diarrhea
E0353	−	0.5	S	O149	1994	K	Diarrhea
E0354	−	0.5	S	O149	1994	K	Diarrhea
E0355	−	0.5	S	O149	1994	K	Diarrhea
E0356	−	0.5	S	O149	1994	K	Diarrhea
E0357	−	0.5	S	O149	1994	K	Diarrhea
E0358	−	0.5	S	O149	1994	K	Diarrhea
E0359	−	0.5	S	O149	1994	K	Diarrhea
E0360	−	0.5	S	O149	1994	K	Diarrhea
E0361	−	0.5	S	O149	1994	K	Diarrhea
E0362	−	0.5	S	O149	1995	K	Diarrhea
E0363	−	0.5	S	O149	1995	K	Diarrhea
E0364	−	0.5	S	O149	1995	K	Diarrhea
E0365	−	0.5	S	O149	1995	K	Diarrhea
E0366	−	0.5	S	O149	1995	K	Diarrhea
E0367	−	0.5	S	O149	1995	K	Diarrhea
E0368	−	0.5	S	O149	1995	K	Diarrhea
E0369	−	0.5	S	O149	1995	K	Diarrhea
E0370	−	0.5	S	O149	1995	K	Diarrhea
E0371	−	0.5	S	O149	1995	K	Diarrhea
E0372	−	0.5	S	O149	1996	K	Diarrhea
E0373	−	0.5	S	O149	1996	K	Diarrhea
E0374	−	0.5	S	O149	1996	K	Diarrhea
E0375	−	0.5	S	O149	1996	K	Diarrhea
E0376	−	0.5	S	O149	1996	K	Diarrhea
E0377	−	0.5	S	O149	1997	K	Diarrhea
E0378	−	0.5	S	O149	1997	K	Diarrhea
E0379	−	0.5	S	O149	1997	K	Diarrhea
E0380	−	0.5	S	O149	1997	K	Diarrhea
E0381	−	0.5	S	O116	2005	F	Diarrhea
E0382	−	0.5	S	O116	2005	F	Diarrhea
E0384	−	0.5	S	O116	2005	F	Diarrhea
E0385	−	0.5	S	O116	2006	F	Diarrhea
E0388	−	0.5	S	O116	2006	F	Diarrhea
E0394	−	0.5	S	O116	2008	F	Diarrhea
E0396	−	0.5	S	O116	2009	F	Diarrhea
E0397	−	0.5	S	O116	2009	F	Diarrhea
E0399	−	0.5	S	O116	2009	F	Diarrhea
E0406	−	0.5	S	O116	2011	F	Diarrhea
E0407	−	0.5	S	O116	2011	F	Diarrhea
E0410	−	0.5	S	O116	2006	F	Diarrhea
E0416	−	0.5	S	O116	2009	F	Diarrhea
E0450	−	0.5	S	O149	2005	C	Diarrhea
E0451	−	0.5	S	OSB9	2006	C	Diarrhea
E0452	−	0.5	S	O149	2006	C	Diarrhea
E0453	−	0.5	S	O149	2006	C	Diarrhea
E0454	−	0.5	S	O149	2006	C	Diarrhea
E0455	−	0.5	S	O149	2006	C	Diarrhea
E0456	−	0.5	S	O149	2006	C	Diarrhea
E0457	−	0.5	S	O149	2006	C	Diarrhea
E0458	−	0.5	S	O149	2006	C	Diarrhea
E0463	−	0.5	S	O149	2006	C	Diarrhea
E0466	−	0.5	S	OSB9	2007	C	Diarrhea
E0473	−	0.5	S	O149	2008	C	Diarrhea
E0474	−	0.5	S	O149	2008	C	Diarrhea
E0480	−	0.5	S	O139	2009	C	Edema disease
E0491	−	0.5	S	O149	2009	C	Diarrhea
E0492	−	0.5	S	O149	2009	C	Diarrhea
E0493	−	0.5	S	O149	2009	C	Diarrhea
E0494	−	0.5	S	O149	2009	C	Diarrhea
E0500	−	0.5	S	OSB9	2010	C	Diarrhea
E0501	−	0.5	S	OSB9	2010	C	Diarrhea
E0503	−	0.5	S	O139	2008	C	Edema disease
E0504	−	0.5	S	OSB9	2010	C	Diarrhea
E0515	−	0.5	S	O149	2005	C	Diarrhea

Strain name	*mcr-1* carriage*	Susceptibility to colistin		O serogroup‡	Characteristic features of isolates		
		MIC (g/mL)	Resistance†		Year	Prefecture	Reported case
E0518	−	0.5	S	OSB9	2005	C	Diarrhea
E0519	−	0.5	S	OSB9	2007	C	Diarrhea
E0541	−	0.5	S	O139	1997	F	Edema disease
E0542	−	0.5	S	O139	1997	F	Edema disease
E0544	−	0.5	S	O139	1998	F	Edema disease
E0545	−	0.5	S	O139	1999	F	Edema disease
E0547	−	0.5	S	O139	2003	F	Edema disease
E0554	−	0.5	S	O149	1997	F	Diarrhea
E0555	−	0.5	S	O149	1998	F	Diarrhea
E0556	−	0.5	S	O149	1998	F	Diarrhea
E0557	−	0.5	S	O149	1998	F	Diarrhea
E0560	−	0.5	S	O149	1999	F	Diarrhea
E0561	−	0.5	S	O149	2000	F	Diarrhea
E0562	−	0.5	S	O149	2003	F	Diarrhea
E0563	−	0.5	S	O149	2003	F	Diarrhea
E0564	−	0.5	S	O149	2004	F	Diarrhea
E0566	−	0.5	S	O149	2005	F	Diarrhea
E0567	−	0.5	S	O149	2008	F	Diarrhea
E0568	−	0.5	S	O149	2008	F	Diarrhea
E0589	−	0.5	S	O149	2012	L	Diarrhea
E0590	−	0.5	S	O149	2012	L	Diarrhea
E0595	−	0.5	S	O116	2012	L	Diarrhea
E0602	−	0.5	S	O139	2012	L	Edema disease
E0603	−	0.5	S	O139	2012	L	Edema disease
E0605	−	0.5	S	O139	2012	L	Edema disease
E0607	−	0.5	S	O116	2012	L	Diarrhea
E0608	−	0.5	S	O116	2012	L	Diarrhea
E0609	−	0.5	S	O116	2012	L	Diarrhea
E0613	−	0.5	S	O116	2012	L	Diarrhea
E0614	−	0.5	S	O116	2012	L	Diarrhea
E0615	−	0.5	S	O116	2012	L	Diarrhea
E0622	−	0.5	S	O139	2012	A	Edema disease
E0623	−	0.5	S	O139	2012	A	Edema disease
E0624	−	0.5	S	O139	2012	A	Edema disease
E0625	−	0.5	S	O139	2012	A	Edema disease
E0626	−	0.5	S	O149	2003	G	Diarrhea
E0627	−	0.5	S	O149	2003	G	Diarrhea
E0630	−	0.5	S	O149	2003	G	Diarrhea
E0631	−	0.5	S	O149	2003	G	Diarrhea
E0653	−	0.5	S	O116	2009	G	Diarrhea
E0660	−	0.5	S	O149	2011	G	Diarrhea
E0676	−	0.5	S	O139	2005	R	Edema disease
E0683	−	0.5	S	O116	2009	G	Diarrhea
E0684	−	0.5	S	O116	2009	G	Diarrhea
E0689	−	0.5	S	O149	2011	G	Diarrhea
E0690	−	0.5	S	O149	2011	G	Diarrhea
E0691	−	0.5	S	O149	2011	G	Diarrhea
E0721	−	0.5	S	O116	2012	E	Diarrhea
E0722	−	0.5	S	O116	2012	E	Diarrhea
E0726	−	0.5	S	O149	2009	E	Diarrhea
E0727	−	0.5	S	O149	2009	E	Diarrhea
E0739	−	0.5	S	O116	2012	E	Diarrhea
E0747	−	0.5	S	O149	2004	E	Diarrhea
E0779	−	0.5	S	O149	2013	Q	Diarrhea
E0780	−	0.5	S	O149	2013	Q	Diarrhea
E0781	−	0.5	S	O149	2013	Q	Diarrhea
E0782	−	0.5	S	O149	2013	Q	Diarrhea
E0865	−	0.5	S	O139	1995	B	Edema disease
E0868	−	0.5	S	O139	1997	B	Edema disease
E0870	−	0.5	S	O139	1999	B	Edema disease
E0899	−	0.5	S	OSB9	2011	B	Diarrhea
E0900	−	0.5	S	OSB9	2011	B	Diarrhea
E0913	−	0.5	S	O149	1999	B	Diarrhea
E0914	−	0.5	S	O149	1999	B	Diarrhea
E0915	−	0.5	S	O149	2000	B	Diarrhea
E0916	−	0.5	S	O149	2001	B	Diarrhea
E0917	−	0.5	S	O149	2001	B	Diarrhea
E0918	−	0.5	S	O149	2004	B	Diarrhea

Strain name	*mcr-1* carriage*	Susceptibility to colistin		O serogroup‡	Characteristic features of isolates		
		MIC (g/mL)	Resistance†		Year	Prefecture	Reported case
E0919	−	0.5	S	O149	2004	B	Diarrhea
E0929	−	0.5	S	O116	2008	B	Diarrhea
E0930	−	0.5	S	O116	2010	B	Diarrhea
E0931	−	0.5	S	O116	2010	B	Diarrhea
E0932	−	0.5	S	O116	2010	B	Diarrhea
E0933	−	0.5	S	O116	2010	B	Diarrhea
E0934	−	0.5	S	O116	2010	B	Diarrhea
E0935	−	0.5	S	O116	2012	B	Diarrhea
E0936	−	0.5	S	O116	2012	B	Diarrhea
E0937	−	0.5	S	O116	2012	B	Diarrhea
E0939	−	0.5	S	O149	2007	V	Diarrhea
E0941	−	0.5	S	O149	2008	V	Diarrhea
E0944	−	0.5	S	O149	2009	V	Diarrhea
E0945	−	0.5	S	O139	2009	V	Edema disease
E0948	−	0.5	S	O139	2010	V	Others/unknown
E0950	−	0.5	S	O139	2012	V	Edema disease
E0963	−	0.5	S	O139	2013	P	Edema disease
E0965	−	0.5	S	O149	2013	P	Diarrhea
E0966	−	0.5	S	O149	2013	P	Diarrhea
E0967	−	0.5	S	O149	2013	P	Diarrhea
E0968	−	0.5	S	O149	2003	H	Diarrhea
E0971	−	0.5	S	OSB9	2005	H	Diarrhea
E0972	−	0.5	S	O149	2005	H	Diarrhea
E0973	−	0.5	S	O149	2005	H	Diarrhea
E0974	−	0.5	S	OSB9	2005	H	Diarrhea
E0977	−	0.5	S	O149	2005	H	Diarrhea
E0980	−	0.5	S	O149	2006	H	Diarrhea
E0981	−	0.5	S	O149	2006	H	Diarrhea
E0982	−	0.5	S	O149	2006	H	Diarrhea
E0983	−	0.5	S	O149	2006	H	Diarrhea
E0985	−	0.5	S	O149	2006	H	Diarrhea
E0988	−	0.5	S	OSB9	2007	H	Diarrhea
E0989	−	0.5	S	O149	2007	H	Diarrhea
E0990	−	0.5	S	O149	2007	H	Diarrhea
E0994	−	0.5	S	OSB9	2007	H	Diarrhea
E0998	−	0.5	S	O149	2008	H	Diarrhea
E0999	−	0.5	S	O149	2008	H	Diarrhea
E1000	−	0.5	S	O149	2009	H	Diarrhea
E1001	−	0.5	S	O149	2009	H	Diarrhea
E1004	−	0.5	S	O149	2009	H	Diarrhea
E1006	−	0.5	S	O149	2009	H	Diarrhea
E1008	−	0.5	S	O116	2009	H	Diarrhea
E1009	−	0.5	S	O149	2009	H	Diarrhea
E1010	−	0.5	S	O149	2009	H	Diarrhea
E1011	−	0.5	S	O116	2009	H	Diarrhea
E1013	−	0.5	S	O149	2009	H	Diarrhea
E1014	−	0.5	S	O149	2010	H	Diarrhea
E1016	−	0.5	S	O149	2010	H	Diarrhea
E1018	−	0.5	S	O149	2010	H	Diarrhea
E1020	−	0.5	S	OSB9	2010	H	Diarrhea
E1021	−	0.5	S	O149	2010	H	Diarrhea
E1022	−	0.5	S	O149	2010	H	Diarrhea
E1023	−	0.5	S	O149	2010	H	Diarrhea
E1024	−	0.5	S	O149	2010	H	Diarrhea
E1025	−	0.5	S	O149	2010	H	Diarrhea
E1026	−	0.5	S	O149	2010	H	Diarrhea
E1027	−	0.5	S	O149	2010	H	Diarrhea
E1028	−	0.5	S	O149	2010	H	Diarrhea
E1029	−	0.5	S	O149	2010	H	Diarrhea
E1030	−	0.5	S	O149	2011	H	Diarrhea
E1033	−	0.5	S	O149	2011	H	Diarrhea
E1034	−	0.5	S	O149	2011	H	Diarrhea
E1038	−	0.5	S	O149	2011	H	Diarrhea
E1045	−	0.5	S	O149	2012	H	Diarrhea
E1049	−	0.5	S	O149	2012	H	Diarrhea
E1051	−	0.5	S	O149	2012	H	Diarrhea
E1054	−	0.5	S	O149	2012	H	Diarrhea
E1058	−	0.5	S	O149	2012	H	Diarrhea

Strain name	*mcr-1* carriage*	Susceptibility to colistin MIC (g/mL)	Resistance†	O serogroup‡	Characteristic features of isolates Year	Prefecture	Reported case
E1060	−	0.5	S	O149	2013	H	Diarrhea
E1077	−	0.5	S	O139	1996	J	Edema disease
E1078	−	0.5	S	O139	1996	J	Edema disease
E1079	−	0.5	S	O139	1996	J	Edema disease
E1080	−	0.5	S	O139	1996	J	Edema disease
E1081	−	0.5	S	O139	1996	J	Edema disease
E1083	−	0.5	S	O139	1997	J	Edema disease
E1084	−	0.5	S	O139	1997	J	Edema disease
E1085	−	0.5	S	O139	1998	J	Edema disease
E1086	−	0.5	S	O139	1999	J	Edema disease
E1087	−	0.5	S	O139	1999	J	Edema disease
E1088	−	0.5	S	O139	1999	J	Edema disease
E1090	−	0.5	S	O139	2001	J	Edema disease
E1092	−	0.5	S	O139	2006	J	Edema disease
E1094	−	0.5	S	O139	2006	J	Edema disease
E1096	−	0.5	S	O139	2008	J	Edema disease
E1098	−	0.5	S	O139	2008	J	Edema disease
E1105	−	0.5	S	O139	2010	J	Edema disease
E1111	−	0.5	S	O139	2014	J	Edema disease
E1117	−	0.5	S	O139	2011	T	Edema disease
E1142	−	0.5	S	O116	2010	W	Diarrhea
E1144	−	0.5	S	O139	2011	W	Edema disease
E1146	−	0.5	S	O139	2013	W	Edema disease
E1147	−	0.5	S	O139	2013	W	Edema disease
E1172	−	0.5	S	O139	2014	E	Others/unknown
E1178	−	0.5	S	OSB9	2005	H	Diarrhea
E1179	−	0.5	S	O139	2012	H	Edema disease
E1181	−	0.5	S	O139	2012	A	Edema disease
E1182	−	0.5	S	O139	2012	A	Edema disease
E1192	−	0.5	S	O139	2013	A	Edema disease
E1193	−	0.5	S	O139	2013	A	Edema disease
E1217	−	0.5	S	OSB9	2008	I	Diarrhea
E1218	−	0.5	S	OSB9	2008	I	Diarrhea
E1226	−	0.5	S	O139	2012	I	Edema disease
E1227	−	0.5	S	O139	2012	I	Edema disease
E1249	−	0.5	S	O139	1999	I	Edema disease
E1250	−	0.5	S	O149	1999	I	Diarrhea
E1251	−	0.5	S	O139	2000	I	Edema disease
E1255	−	0.5	S	O149	1999	I	Diarrhea
E1256	−	0.5	S	O149	1999	I	Diarrhea
E1286	−	0.5	S	OSB9	2003	I	Diarrhea
E1313	−	0.5	S	O149	2014	I	Diarrhea
E1387	−	0.5	S	O139	2010	D	Edema disease
E1390	−	0.5	S	O116	2010	D	Diarrhea
E1392	−	0.5	S	O116	2011	D	Diarrhea
E1396	−	0.5	S	O149	2011	D	Diarrhea
E1397	−	0.5	S	O116	2011	D	Diarrhea
E1398	−	0.5	S	O116	2011	D	Diarrhea
E1400	−	0.5	S	O116	2011	D	Diarrhea
E1412	−	0.5	S	O116	2011	D	Diarrhea
E1413	−	0.5	S	O116	2011	D	Diarrhea
E1414	−	0.5	S	O116	2012	D	Diarrhea
E1419	−	0.5	S	O149	2012	D	Diarrhea
E1420	−	0.5	S	O149	2012	D	Diarrhea
E1425	−	0.5	S	O139	2012	D	Edema disease
E1428	−	0.5	S	O149	2012	D	Diarrhea
E1429	−	0.5	S	O139	2012	D	Edema disease
E1438	−	0.5	S	O116	2013	D	Diarrhea
E1439	−	0.5	S	O139	2013	D	Edema disease
E1440	−	0.5	S	O116	2013	D	Diarrhea
E1441	−	0.5	S	O149	2013	D	Diarrhea
E1449	−	0.5	S	O149	2014	D	Diarrhea
E1450	−	0.5	S	O139	2014	D	Edema disease
E1453	−	0.5	S	O116	2014	D	Diarrhea
E1515	−	0.5	S	O139	2013	P	Edema disease
E0242	−	0.25	S	O139	1997	S	Edema disease
E0516	−	0.25	S	OSB9	2005	C	Diarrhea
E0551	−	0.25	S	O139	2009	F	Edema disease

Strain name	*mcr-1* carriage*	Susceptibility to colistin		O serogroup‡	Characteristic features of isolates		
		MIC (g/mL)	Resistance†		Year	Prefecture	Reported case
E0552	−	0.25	S	O149	1996	F	Diarrhea
E0553	−	0.25	S	O149	1996	F	Diarrhea
E0558	−	0.25	S	O149	1999	F	Diarrhea
E0559	−	0.25	S	O149	1999	F	Diarrhea
E0586	−	0.25	S	O139	2012	L	Edema disease
E0718	−	0.25	S	O139	2013	E	Edema disease
E0719	−	0.25	S	O139	2013	E	Edema disease
E0720	−	0.25	S	O139	2013	E	Edema disease
E0728	−	0.25	S	O149	2007	E	Diarrhea
E0729	−	0.25	S	O149	2007	E	Diarrhea
E0731	−	0.25	S	O149	2006	E	Diarrhea
E0732	−	0.25	S	O149	2006	E	Diarrhea
E0740	−	0.25	S	O149	2007	E	Diarrhea
E0741	−	0.25	S	O149	2007	E	Diarrhea
E0869	−	0.25	S	O139	1998	B	Edema disease
E0964	−	0.25	S	O139	2013	P	Edema disease
E1015	−	0.25	S	O149	2010	H	Diarrhea
E1075	−	0.25	S	O139	1996	J	Edema disease
E1076	−	0.25	S	O139	1996	J	Edema disease
E1082	−	0.25	S	O139	1997	J	Edema disease
E1089	−	0.25	S	O139	2000	J	Edema disease

*+, *mcr-1*-positive isolate; −, *mcr-1*–negative isolate.
†R, colistin-resistant isolate; S, colistin-susceptible isolate.
‡SB9, *Shigella boydii* type 9.
